# *SPO24* Is a Transcriptionally Dynamic, Small ORF-Encoding Locus Required for Efficient Sporulation in *Saccharomyces cerevisiae*

**DOI:** 10.1371/journal.pone.0105058

**Published:** 2014-08-15

**Authors:** Sara Hurtado, Karen S. Kim Guisbert, Erik J. Sontheimer

**Affiliations:** Department of Molecular Biosciences, Northwestern University, Evanston, Illinois, United States of America; University of Cambridge, United Kingdom

## Abstract

In *Saccharomyces cerevisiae*, meiosis and sporulation are highly regulated responses that are driven in part by changes in RNA expression. Alternative mRNA forms with extended 5′ UTRs are atypical in *S. cerevisiae*, and 5′ extensions with upstream open reading frames (uORFs) are even more unusual. Here we characterize the gene *YPR036W-A*, now renamed *SPO24*, which encodes a very small (67-amino-acid) protein. This gene gives rise to two mRNA forms: a shorter form throughout meiosis and a longer, 5′-extended form in mid-late meiosis. The latter form includes a uORF for a 14-amino-acid peptide (Spo24^u14^). Deletion of the downstream ORF (dORF) leads to sporulation defects and the appearance of pseudohyphae-like projections. Experiments with luciferase reporters indicate that the uORF does not downregulate dORF translation. The protein encoded by the dORF (Spo24^d67^) localizes to the prospore membrane and is differentially phosphorylated during meiosis. Transcription of the 5′-extended mRNA in mid-meiosis depends upon the presence of two middle sporulation elements (MSEs). Removal of the MSEs severely inhibits the mid-meiotic appearance of the 5′-extended mRNA and limits the ability of plasmid-borne *SPO24* to rescue the sporulation defect of a *spo24*Δ mutant, suggesting that the 5′-extended mRNA is functionally important. These results reveal Spo24^d67^ as a sporulation-related factor that is encoded by a transcriptionally dynamic, uORF-containing locus.

## Introduction

Meiosis enables the formation of haploid gametes from a diploid progenitor. In the budding yeast *Saccharomyces cerevisiae*, meiosis is followed by spore formation and depends upon a highly ordered transcriptional cascade that is triggered by specific nutritional and genetic conditions [1]–[4]. Budding yeast meiosis is driven in part by three transcriptional phases [5]. The early phase includes genes important for the response to nutrient stress and initiation of meiosis I (MI), which includes DNA replication and recombination. The middle phase includes the end of MI as well as meiosis II (MII). During the late transcriptional phase, synthesis of the spore wall occurs and the mature ascus is generated. Checkpoints monitor the success of critical steps within the pathway, enforcing correct completion before allowing the cell to progress [3].

Several transcription factors are involved in turning on the early, middle and late genes of sporulation. Early genes are activated at their URS1 (Upstream Regulatory Sequence 1) site by Ume6 [6], which is converted from a negative regulator to a positive regulator by Ime1 [2], [6], [7]. The majority of midsporulation genes are regulated by a small sequence called the midsporulation element (MSE) [8], to which the transcription factor Ndt80 binds to activate the associated genes. These Ndt80-regulated genes are involved in a variety of processes related to progression through meiotic divisions, formation of the anaphase-promoting complex, and sporulation [3], [5], [9]–[11]. Ndt80, in collaboration with other factors, also helps to regulate the smaller subset of late genes induced after MII [12].

During and after the late phase of expression, the four haploid nuclei are encapsulated into spores [13]. Spore formation begins with recruitment of vesicles to the cytoplasmic sides of the four spindle pole bodies of the haploid nuclei [14], [15]. These vesicles flatten to form the prospore membrane [15]. Throughout MII, the four prospore membranes expand to fully engulf the nuclear lobes that are anchored to the spindle pole bodies [16]. At the end of nuclear division, the encapsulation is complete and the daughter nuclei are each contained within an individual cytoplasm, creating four separate prospores with double membranes [13]. Maturation of the spores occurs through synthesis of the spore wall [17] and the breakdown of the outer membrane [18], [19]. After formation of the spore wall, the anucleate mother cell remodels to form the encapsulating ascus [20]–[22].

Many meiosis- and sporulation-defective mutants have been identified, initially through a classical genetic screen [23] and more recently through the analysis of genome-wide collections of deletions at long (>80–100 codons) open reading frames (ORFs) [5], [24]–[28]. More than 300 genes have been found to be essential for sporulation [24], [26], [27], and hundreds more are differentially expressed [5], [25], [28]. More recently, meiotic expression analysis has extended beyond annotated ORFs to include 5′ and 3′ untranslated regions (UTRs), small unannotated ORFs, and intergenic regions [29]–[31]. These analyses have demonstrated that the meiotic transcriptome is unexpectedly dynamic, not only in terms of gene activation and repression, but also in terms of transcript architecture itself [29]–[31]. For hundreds of transcripts, 5′- and 3′-terminal boundaries change as meiosis and sporulation progress. For many such loci, the altered mRNAs are predicted to give rise to different protein isoforms; many other cases include the appearance of mRNAs with small upstream ORFs (uORFs) in addition to the canonical, longer, annotated ORF (downstream ORF, or dORF). Ribosome profiling has shown that the meiotic gene regulatory program is replete with examples of translational control, including uORF translation that is sometimes, though not always, accompanied by reduced ribosome occupancy of the associated dORF [30]. In most cases, the effect of translational control on the meiotic pathway itself is unknown.

A growing body of evidence suggests that in eukaryotes, uORF-mediated translational control may serve as a common regulatory mechanism for protein expression. For example, in mice, the mRNA encoding CCAAT/enhancer binding protein Beta (C/EBPβ) yields different protein isoforms depending on the presence or absence of a uORF [32]. An especially well-known case is the yeast gene *GCN4*, which generates an mRNA that includes four uORFs [33] that control Gcn4 translation through a process called reinitiation [34]. *GCN4* encodes a transcription factor that regulates gene expression in response to amino acid starvation. Under non-stressed conditions there is an abundance of eIF2-GTP-tRNA(i) ternary complex (TC) for binding and activation of the 40S ribosomal subunit. As a result, *GCN4* uORF1 is efficiently translated, and then the 40S subunit resumes scanning. The high TC levels enable the 40S subunit to acquire a new TC in time to translate uORF4, termination of which limits ribosome access to the *GCN4* dORF [33], [35], [36]. During amino acid starvation, TC levels drop, limiting the frequency with which the scanning 40S ribosomal subunit reinitiates translation at the inhibitory uORF4, enabling subsequent Gcn4 translation. Despite these examples, in most cases of uORF-containing mRNAs expressed during yeast meiosis, the effect of translational control (if any) on the meiotic pathway itself is unknown.

We previously identified *YPR036W-A* as a largely uncharacterized yeast gene that encodes a 67-amino-acid polypeptide and that exhibits a striking 5′-UTR extension during mid-meiosis [29]. Here we analyze this gene and show that its deletion leads to a sporulation-defective phenotype, and we have therefore renamed it *SPO24*. The sporulation defect is accompanied by the appearance of pseudohyphae-like projections. We find that a fifteen-codon (including the stop codon) uORF that is present in the extended mRNA form does not downregulate translation from the dORF. Nonetheless, deletion of regulatory sequences necessary for the expression of the 5′-extended mRNA leads to defective sporulation. The protein encoded by the dORF (Spo24^d67^) is differentially phosphorylated during meiosis and localizes to the prospore cortex. Our results define a transcriptionally dynamic locus that encodes a previously unrecognized and unusually small sporulation factor in yeast.

## Results

### A 5′-Extended *YPR036W-A* Transcript is Activated in Middle and Late Meiosis

**Figure 1:**
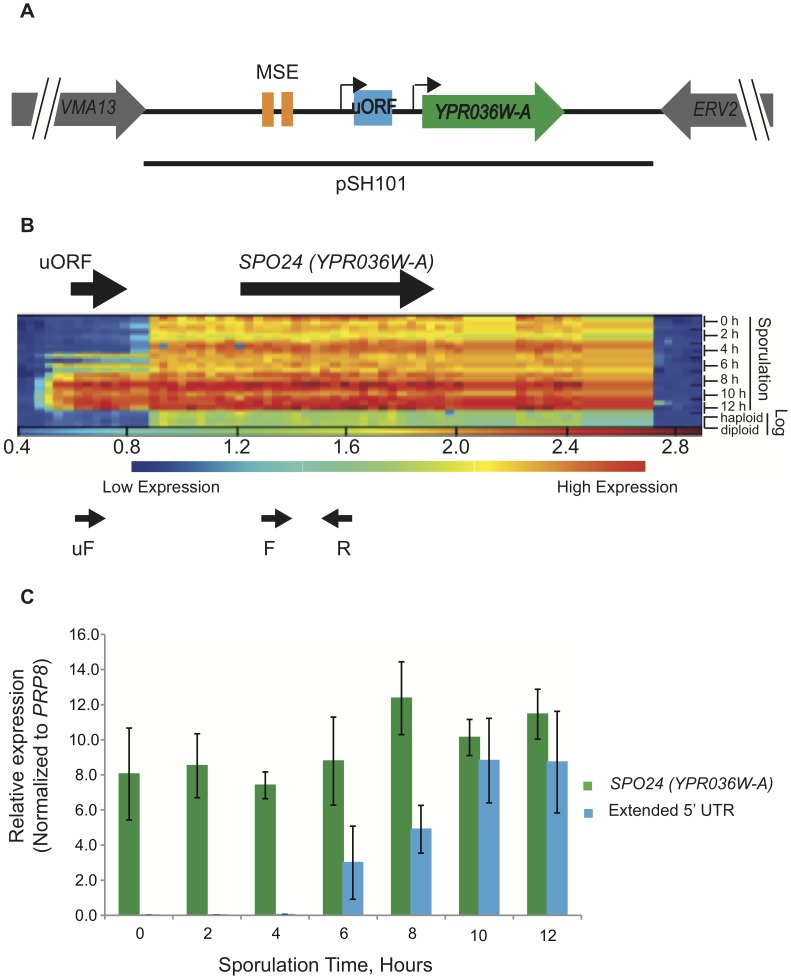
*YPR036W-A* expresses two mRNA forms. (**A**) Map of the *YPR036W*-*A* locus in *S. cerevisiae*. The region upstream of the 204-nt *YPR036W-A* dORF includes a 45-nt uORF that is present in a longer form of expressed mRNA that is induced in mid-meiosis. Further upstream are two consensus Ndt80 transcription factor binding sites (middle sporulation elements, or MSEs). The line denotes the boundaries of the genomic fragment included in the rescue plasmid pSH101. Black arrows mark the approximate transcription start sites, as mapped through the tiling array signal and confirmed by 5′ RACE. (**B**) Heat map of tiling array data showing *YPR036W-A* expression during meiosis. Genomic coordinates along the *YPR036W-A* locus correspond to the horizontal axis. The array signals (from three separate cultures for each time point) are stacked vertically with the beginning of meiosis at the top, and with sporulation times indicated to the right of the heat map. The bottom six layers are from log-phase haploid and diploid cells. The positions of the uORF and dORF are given by the upper arrows. After ∼6 hours of sporulation a meiosis-specific RNA is induced. Small arrows underneath represent primer-binding sites for reverse (R), forward (F), and upstream forward (uF) primers. (**C**) qRT-PCR analyses using the primers depicted in (B) show expression patterns consistent with the array data, confirming the presence of a longer RNA. Moreover the analysis indicates that the extended signal detected on the arrays is a longer RNA that is contiguous into the dORF, and does not simply reflect expression of a distinct, neighboring transcript that abuts a shorter *YPR036W-A* mRNA. Error bars represent the standard error of the mean (SEM) from three biological replicates.

We previously used custom-designed, high-resolution tiling arrays to determine the expression and transcript architecture of coding and noncoding RNAs in both log-phase and sporulating *S. cerevisiae* cells from the SK1 strain background [29]. We noted the existence of numerous transcripts with extended 5′ UTRs that appear during meiosis. Included in this set was the gene *YPR036W-A* (Figure 1A), which exhibits a sharp shift to a longer transcript at mid-meiosis (Figure 1B). This longer transcript, which adds 100+/−5 nts to the 5′ UTR (relative to the mRNA that is expressed in early meiosis), persists through the remainder of meiosis and sporulation. The extended mRNA includes a single fifteen-codon (including the stop codon) uORF that is not detectably expressed during early meiosis or log phase. The annotated dORF encoded by *YPR036W-A* is small (68 codons), and the function of the encoded protein is unknown. Because the *YPR036W-A* dORF is less than 80–100 codons, it was excluded from ORF deletion and GFP fusion collections – and therefore from genome-wide screens and analyses using these collections – reported to date. *YPR036W-A* was originally identified in 2001 in a screen for transcripts regulated by the transcription factor Pdr1 [37], but very little else is known about its expression and function. Because it was not included in previous genome-wide screens for ORF deletions with meiotic-specific phenotypes, its roles during meiosis and sporulation, if any, are unknown.

We confirmed the expression of a 5′-extended meiotic form of the *YPR036W-A* mRNA using qRT-PCR (normalized to *PRP8* mRNA, Figure 1C), primer extension (Figure S1A) and 5′ RACE analysis (Figure S1B). The qRT-PCR and 5′ RACE analyses using a reverse primer (“R” in Figure 1B) within the dORF revealed that the 5′-extended signal detected by our microarray analysis (Figure 1B) reflects a transcript that is contiguous into the dORF (Figures 1C and S1B). qRT-PCR experiments with RNA samples taken every two hours during a twelve-hour meiotic time course, and using forward and reverse primers from within the *YPR036W-A* dORF, showed that dORF-encoding mRNAs (regardless of 5′ UTR length) are expressed throughout meiosis (Figure 1C, light bars). In contrast, when we performed qRT-PCR on the same samples using a forward primer specific for the 5′-extended region, the longer transcript was detected only at the mid-meiotic time point (6 hours) and thereafter (Figure 1C, dark bars), in agreement with the microarray data. The induction of the long form in mid-meiosis is not accompanied by a loss of the shorter form, as both transcripts can be detected later in meiosis by primer extension and 5′ RACE (Figures S1A and S1B). We conclude that *YPR036W-A* encodes two mRNA forms: a short form that is expressed throughout meiosis, and a 5′-extended, uORF-containing form that is restricted to middle and late meiosis.

### The *YPR036W-A* dORF Is Required for Efficient Sporulation

**Figure 2:**
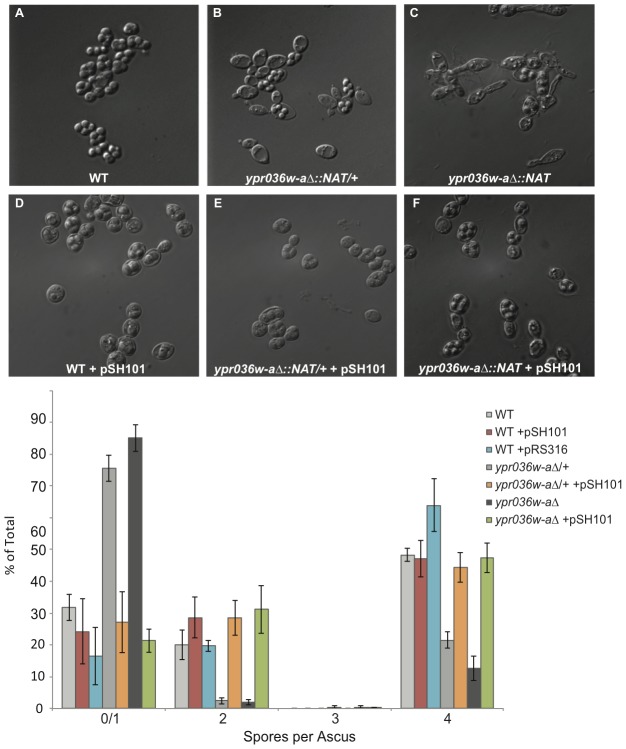
*YPR036W-A* (*SPO24*) is required for efficient sporulation. (**A–C**) Differential interference contrast (DIC) images of sporulated wildtype (DKB98) yeast cells (A), the *spo24*Δ/+ heterozygous deletion derivative (B), and the *spo24*Δ homozygous deletion derivative (C), respectively. The *spo24*Δ/+ heterozygote exhibits decreased sporulation efficiency and a more elongated morphology (B). Both the sporulation defect and the pseudohyphae-like morphology are exacerbated in the *spo24*Δ homozygote (C), suggesting a gene dosage effect on the phenotypes. (**D–F**) As in A–C, respectively, but with cells that harbor the *SPO24*-containing, *CEN* rescue plasmid pSH101 (see Figure 1A). The rescue plasmid abolishes the sporulation efficiency defect as well as the elongated cellular morphology. (**G**) Quantification of sporulation efficiency, as measured by the number of spores per ascus (n = 200 for each of three biological replicates) after 40 hours on solid sporulation medium. Complementation of the *spo24* deletion with the pSH101 plasmid results in partial rescue the sporulation defect.

To address the function of *YPR036W-A*, we used homologous recombination in the SK1-derived strain DKB98 to replace the dORF with a selectable marker (*NAT*). *ypr036w-a*Δ haploid spores readily formed colonies on solid media, indicating that the dORF is not essential for germination or viability. Furthermore, we observed no growth defect of the *ypr036w-a*Δ haploid cells (relative to DKB98) in liquid media (Figure S2). However, *ypr036w-a*Δ homozygous diploid cells were partially sporulation-defective, with 15%±11.5% (n = 600) of cells giving rise to tetrads after twelve hours of sporulation, compared to 68.5%±8.2% (n = 600) for the isogenic wildtype control strain (*p*<0.01, Student's t-test) (Figure 2A, C and G). Of the *ypr036w-a*Δ cells that did not sporulate, the majority (64%, n = 100) were tetranucleate (Figure S3) and an additional 16% were binucleate, as indicated by DAPI staining. This suggests that most *ypr036w-a*Δ diploid cells successfully initiate meiosis, and that most of those complete MI and MII but not sporulation. Interestingly, the *ypr036w-a*Δ/+ heterozygote (Figure 2B) also exhibited a sporulation defect [22.5%±2.1% (n = 600) of the cells yielded asci with four spores after twelve hours], suggesting a gene dosage dependency. Transformation of a complementing *CEN* plasmid (pSH101, Figure 1A) partially rescues the sporulation efficiency defect, with 42.2%±3.5% (*p*<0.05) and 49.6%±1.8% (*p*<0.01) of plasmid-bearing cells successfully completing sporulation in the *ypr036w-a*Δ heterozygous and homozygous backgrounds, respectively (Figure 2E–G). Overexpression of *YPR036W-A* [via introduction of pSH101 into wild-type cells (Figure 2D and G)] did not significantly inhibit sporulation (63.5%±8.2% tetrads) (Figure 2A and G). Based on the sporulation efficiency defects in the *ypr036w-a*Δ mutants, we have renamed this gene *SPO24*.

Sporulation of wild-type diploid cells usually yields four discrete spores, with the mother cell having collapsed to form the mature ascus (Figure 2A). When one copy of the *SPO24* dORF is deleted, the asci no longer appear fully mature and collapsed, but rather appear to have a void volume between the ascus wall and the spores (Figure 2B). Morphological changes that include a “pseudohyphae-like,” extended cell shape are also apparent in the asci generated by *spo24*Δ/+ heterozygous diploids (Figure 2B), and this phenotype is even more pronounced with the *spo24*Δ homozygotes (Figure 2C). Rescue with pSH101 ameliorated both the sporulation efficiency and pseudohyphae-like phenotypes (Figure 2E–F). Intriguingly, the *spo24*Δ diploid strain (and, to a lesser extent, the *spo24*Δ/+ heterozygous strain) also shows signs of an elongated, pseudohyphae-like morphology during vegetative growth (Figure S4), consistent with the detectable levels of *SPO24* expression (Figure 1B and Figure S1A).

### The *SPO24* uORF Modestly Increases Translational Efficiency of the *SPO24* dORF

**Figure 3:**
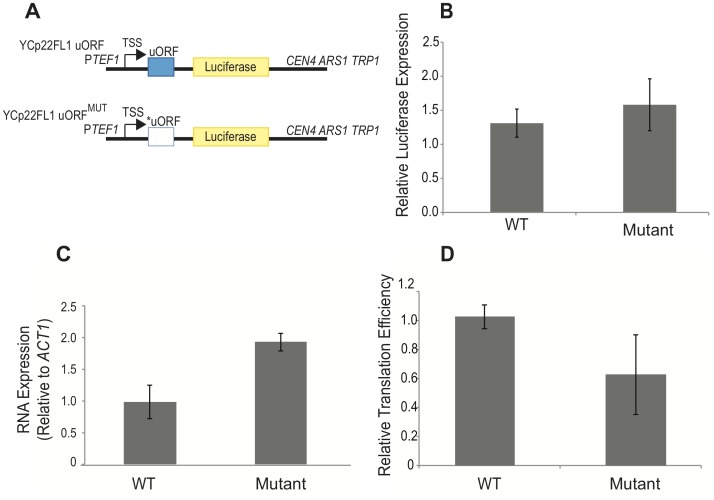
Modest stimulation of luciferase expression by the *SPO24* uORF. (**A**) Diagram of the luciferase reporter constructs. The YCp22FL1 reporter carrying the wild-type *SPO24* uORF is shown at the top. A mutant in which the uORF AUG translation initiation codon was changed to a CUG codon was also generated (below, marked with an asterisk). Reporter expression is driven by the P*_TEF1_* promoter. (**B**) Plasmids in (A), as well as the control FL' plasmid, were introduced into (strain) and maintained in the absence of Trp, and lysates from these cells were subjected to luciferase assays. The graph shows luciferase expression in the presence of the wild-type uORF (WT) and the mutated uORF, normalized to expression from the control FL' plasmid. (**C**) qRT-PCR data of luciferase mRNA levels, normalized to those of *ACT1*. (**D**) Translational efficiency calculated from protein levels [luciferase expression, (B)] and mRNA levels [RT-qPCR data, (C)] shows a small increase in translational efficiency relative to wild type when the uORF is present. In B–D, error bars represent SEM calculated from three biological replicates.

The gene dosage-dependent effect of the *SPO24* dORF on sporulation efficiency suggests that Spo24^d67^ expression or activity is tightly controlled during meiosis. We hypothesized that the presence of the uORF in the 5′-extended mRNA that is induced in mid-meiosis could contribute to this control, since uORFs sometimes (but not always) limit translation of dORFs by preventing scanning translation initiation complexes from reaching the dORF initiation codon [36]. To test this hypothesis, we inserted 95 nts from the *SPO24* 5′-extended region (including the uORF, along with 43 and 7 nts of 5′- and 3′-flanking sequence, respectively) into the yeast-specific luciferase reporter construct yCP22FL1 [38] to generate yCP22FL1-uORF (Figure 3A). We also generated a control construct (yCP22FL1-uORF^MUT^) that was identical except for an AUG→CUG mutation in the uORF initiation codon (Figure 3A). We also used the empty plasmid yCP22FL' (i.e., containing no uORF upstream of luciferase) for standardization of luciferase expression and activity. In all cases, reporter expression was driven by the *TEF1* promoter. Plasmids were transformed into wildtype W303A yeast, and following growth and expression, cells were lysed and assayed for mRNA levels as well as luciferase activity. Normalized to yCP22FL', yCP22FL1-uORF had a relative luciferase expression of 1.3±0.2 (Figure 3B), suggesting that the *SPO24* uORF can have at most a mild effect on expression. Mutation of the uORF's AUG initiation codon in yCP22FL1-uORF^MUT^ led to relative expression (again normalized to that of yCP22FL') of 1.6±0.4 (Figure 3B), which is marginally different from that of the unmutated plasmid (*p* = 0.37, Student's t-test).

Luciferase expression is a function of both RNA accumulation as well as translation. It therefore remained possible that the uORF insert affected both in opposing manners, masking any translation-specific effect. We therefore assayed reporter transcript levels by qRT-PCR, normalizing to mRNAs expressed from a control housekeeping gene (*ACT1*). We found that mutation of the uORF start codon led to an ∼2-fold increase in steady-state mRNA levels (Figure 3C) relative to the unmutated uORF-containing construct, perhaps by limiting the ability of the uORF to direct the reporter mRNA into the nonsense-mediated-decay pathway [39]. The translation efficiency, defined as the ratio of protein expression (as reflected in luciferase activity) to mRNA levels (see Methods), decreased slightly when the start codon was abolished (Figure 3D), indicating that the intact uORF has a modest stimulatory effect on translation. We conclude that the *SPO24* uORF is unlikely to play an inhibitory role in Spo24^d67^ translation. These experiments, which for technical reasons were done with vegetatively growing cultures, do not by themselves exclude the possibility that the *SPO24* uORF does inhibit Spo24^d67^ translation specifically during meiosis, when the 5′-extended form of *SPO24* mRNA is transcribed. Nonetheless, recent ribosome profiling experiments in staged meiotic cultures have revealed numerous cases in which increased uORF translation is positively correlated with ribosome density on the associated dORF, and the *SPO24* uORF and dORF were in this category [30].

### Two *SPO24* MSE Consensus Sites are Required for Efficient Sporulation

To further understand the regulatory basis for Spo24^d67^ expression, we examined the sequences upstream of the *SPO24* transcribed regions and identified two candidate middle sporulation elements (MSEs), which in many other genes serve as binding sites for the meiosis-specific transcription factor Ndt80. This DNA-binding protein is the major transcription factor driving the expression of genes that, like the 5′-extended form of *SPO24*, are activated in mid-meiosis. To examine the role of the MSEs, we used site-directed mutagenesis to create deletion mutations in both putative MSEs in the *SPO24* rescue plasmid (Figure 1A), generating pSH110(MSEΔ). We carried out qRT-PCR analyses of RNA samples from a meiotic time course, using the reverse and upstream forward primers (Figure 1B) that detect expression of the 5′-extended *SPO24* mRNA. When *spo24*Δ cells were complemented with the *SPO24* plasmid pSH101, the 5′-extended mRNA was readily detected six hours into meiosis and thereafter (Figure S5A), consistent with our previous results (Figure 1C). In contrast, removal of the MSEs from the rescue plasmid led to a drastic reduction in levels of the 5′-extended mRNA (Figure S5A). Surprisingly, overall levels of dORF-containing transcripts were unchanged or slightly higher (up to ∼2.5-fold, depending on the time point) with the pSH110(MSEΔ) rescue plasmid (Figure S5B), suggesting that the loss of the 5′-extended transcripts is compensated for by other transcriptional or post-transcriptional effects on mRNA accumulation. These results indicate that the MSEs are indeed important for induction of the 5′-extended *SPO24* mRNA during meiosis.

**Figure 4:**
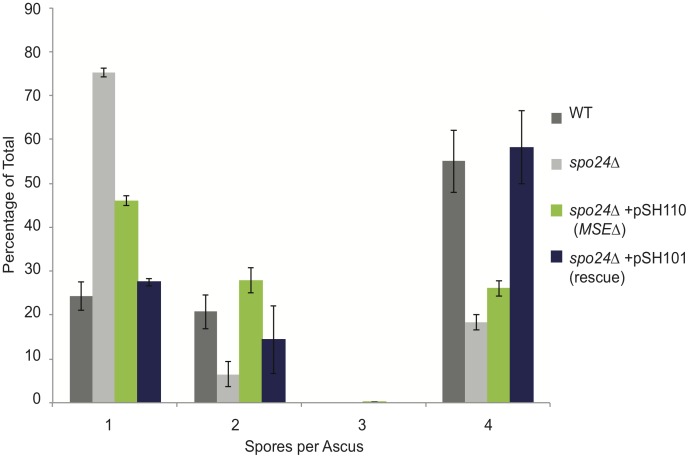
Deletion of the Ndt80 binding sites from the *SPO24* promoter decreases sporulation efficiency. The data show wildtype and *spo24*Δ sporulation efficiencies in comparison with those of the deletion strain carrying a *SPO24* wildtype rescue plasmid (pSH101) and a derivative with its two MSEs deleted (pSH110). The wildtype plasmid fully rescues the *spo24*Δ sporulation defect, whereas the ΔMSE derivative does not.

We next tested the requirement for the MSEs during meiosis by comparing sporulation efficiencies of the *spo24*Δ mutant strain carrying pSH110(MSEΔ) to those of the same strain carrying either the wildtype rescue plasmid (pSH101) or no rescue plasmid, as well as the parental *SPO24* strain. In this experiment, after 41 hours on sporulation plates, 55%±7.1% of wildtype DKB98 cells had formed tetrads (Figure 4). When analyzed in parallel, only 18.3%±1.8% of *spo24*Δ cells completed sporulation (*p* = 0.065). Introduction of the complementing *SPO24* plasmid pSH101 into the *spo24*Δ cells rescues sporulation back to wildtype levels (58.2%±8.3%) (Figure 4). Complementation is mostly lost when the MSEs are deleted, with only 26%±7.0% of the pSH110(MSEΔ)-transformed *spo24*Δ cells forming tetrads (*p* = 0.02) (Figure 4). These results indicate that the MSEs are important for full *SPO24* function during meiosis, despite the fact that their deletion does not affect overall dORF-containing mRNA accumulation (see discussion). Although pSH110(MSEΔ) failed to restore efficient tetrad formation to *spo24*Δ cells, we did observe an increase in dyads (asci containing two spores), with dyad frequencies rising from 6.5%±2.8% to 27.8%±12% in the *spo24*Δ and pSH110(MSEΔ) strains, respectively (Figure 4). The low frequency of dyad formation in the *spo24*Δ cells may be due in part to a defect in meiotic entry or early meiosis. The increase in dyad but not tetrad frequency upon introduction of pSH110(MSEΔ) may reflect a rescue of Spo24^d67^ function early in the pathway, but then a failure to rescue later Spo24^d67^ functions due to the loss of the 5′-extended transcript. Interestingly, pSH110(MSEΔ)-rescued, sporulated *spo24*Δ cells did not exhibit the pseudohyphae-like phenotype (Figure S5C), suggesting that the 5′-extended mRNA does not play a role in the morphological aspects of *SPO24* function.

### *SPO24* Encodes a Phosphoprotein that is Conserved in Other Yeasts

**Figure 5:**
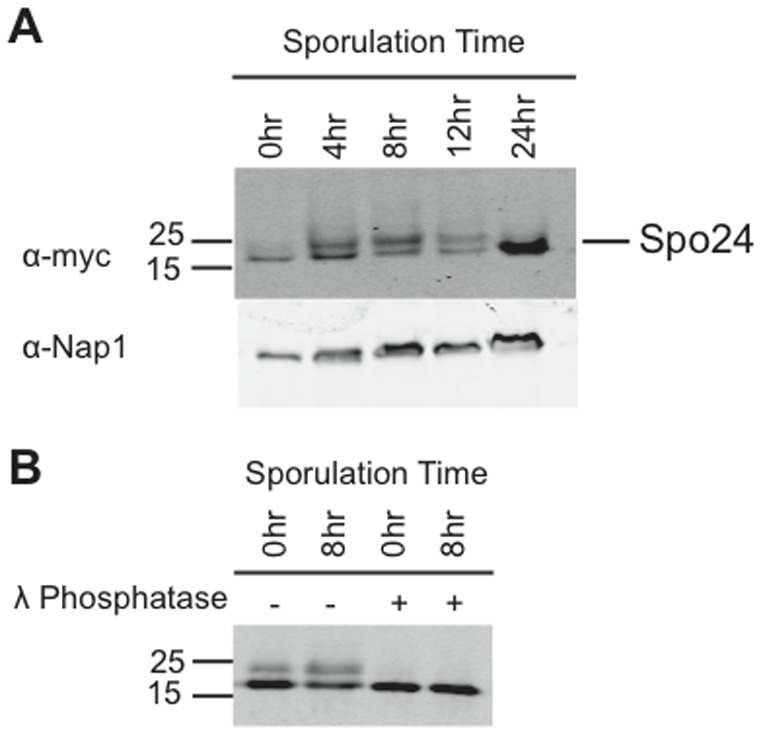
Western analysis of epitope-tagged Spo24^d67^ protein. (**A**) Protein samples from a sportulation time course were subjected to Western analysis with antibodies against a 9-Myc tag fused to the C-terminus of Spo24^d67^. The blots reveal a protein of the expected molecular weight (21.5 kDa), indicating that the Spo24^d67^ protein is expressed throughout meiosis. Nap1 was also analyzed as a loading control. The protein appears as a doublet at multiple time points, suggesting the existence of a posttranslationally modified form. (**B**) The upper band of the Spo24^d67^ doublet is susceptible to λ phosphatase treatment. Both Spo24 bands persist after mock digestion, whereas the upper band is abolished by λ phosphatase, indicating that the upper band is a phosphorylated isoform.

Protein expression from the *SPO24* locus has not been well characterized. To address this issue, we used homologous recombination to introduce a 9xMyc tag to the C-terminus of the *SPO24* dORF. Unlike deletion of the *SPO24* dORF, the addition of the tag had no effect on sporulation efficiency (Figure S6), suggesting that the tag does not compromise *SPO24* function. Protein samples from a meiotic time course were analyzed by probing western blots with anti-myc antibody, and a protein of the predicted size (21.5 kDa) was detected throughout meiosis (Figure 5). This protein was not detected in the untagged wildtype cells (Figure S7), indicating that the detection was specific.

**Figure 6:**
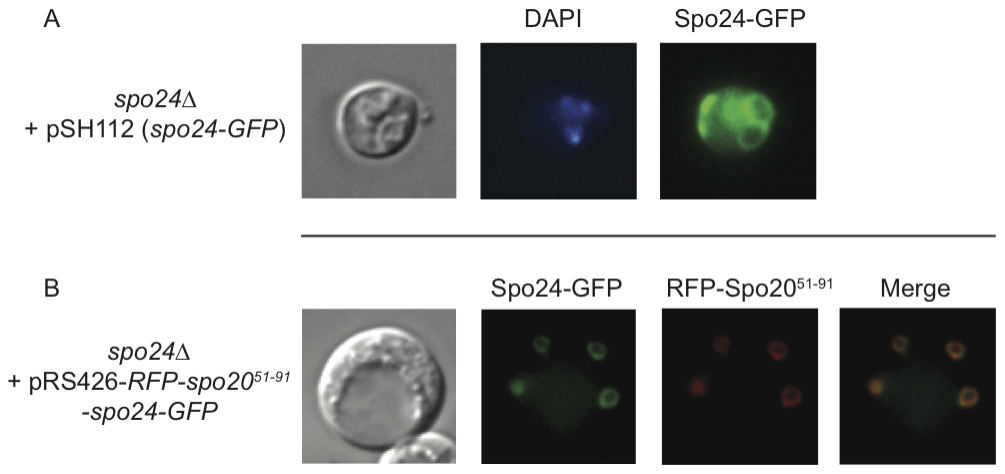
Spo24^d67^ localizes to the prospore cortex. (**A**) *spo24*Δ cells carrying a plasmid (pSH112) expressing Spo24-GFP were sporulated on SPM plates for 24 hours. GFP fluorescence microscopy (right panel) revealed ring-like structures that appear to correspond with spore locations in the DIC image left panel). (**B**) As in (A), except that the cells carrying a plasmid (pRS426_RFP_ Spo20^51–91^ Spo24_GFP) expressing both RFP-Spo20^51–91^ (a prospore marker) and Spo24-GFP. The GFP ring-like structures co-localize with the prospore marker protein (in red, merge represented by yellow), suggesting that Spo24 is associated with the prospore cortex.

The western blots revealed not only the expected 21.5 kDa protein, but also a slightly slower-migrating band that was most pronounced during mid-meiosis (Figure 5). The slightly retarded mobility of the upper band suggested that it may be a post-translationally modified (e.g., phosphorylated) form of the Spo24^d67^ protein. To test whether the upper band was a phosphoprotein, we subjected protein samples from the epitope-tagged strain to lambda phosphatase digestion, in parallel with a mock-digested control. The treated samples were then analyzed by anti-myc western blots (Figure 6B). Lambda phosphatase treatment collapsed the doublet to single band, indicating that the upper band of the doublet is a phosphorylated form of the Spo24^d67^ protein (Figure 6B). The Spo24^d67^ amino acid sequence includes several consensus phosphorylation sites (Figure S8), and inspection of publicly available *S. cerevisiae* phosphoproteomic datasets confirms these sequences as sites of phosphorylation [40]–[43], consistent with our findings. We conclude that the Spo24^d67^ protein is expressed and differentially phosphorylated during meiosis. Aside from the apparent phosphorylation sites, the Spo24^d67^ amino acid sequence and conservation reveals no known domains that would suggest particular cellular or biochemical functions.

### The Spo24^d67^ Protein Localizes to the Prospore

The subcellular localization of a protein can provide important insight into its function. To examine Spo24^d67^ localization, we used gap repair to fuse the coding sequence for green fluorescent protein (GFP) to the C-terminus of the *SPO24* dORF in the pSH101 rescue plasmid. Sporulation assays demonstrated that the GFP tag had no detrimental effect on the rescue of the *spo24*Δ sporulation defect, indicating that the GFP-tagged protein is functional (Figure S6).

We next used fluorescence microscopy to examine the subcellular distribution of GFP-tagged Spo24^d67^ protein in sporulated cultures. Ring-like structures were evident in *spo24*Δ cells carrying the Spo24^d67^-GFP-expressing plasmid (Figure 6A). We observed these rings in cells that contained no spores that were discernable by differential interference contrast microscopy, as well as those with visible spores, This suggests that the Spo24^d67^ localization pattern is established early in sporulation, such as during prospore membrane formation [18]. We obtained a plasmid expressing a red fluorescent protein (RFP)-tagged prospore marker (Spo20^51–91^) [44] and modified it by the insertion of our *SPO24*-GFP construct. We introduced this plasmid into wildtype DKB98, and the transformed cells were imaged for RFP and GFP fluorescence. We observed clear co-localization of Spo24^d67^-GFP with the RFP-Spo20^51–91^ [44] prospore marker (Figure 6B), confirming that the Spo24^d67^-GFP rings reflect localization to the prospore membrane.

## Discussion

We report the characterization of *SPO24* (previously *YPR036W-A*), a gene that encodes a novel and unusually small (67 amino acids) sporulation factor and that exhibits a dynamic expression pattern during meiosis. As noted previously by us [29] and others [30], a *SPO24* 5′-extended mRNA is induced at mid-meiosis, and the 5′-extension includes a uORF with the potential to encode a 14-amino-acid peptide. Ribosome profiling data indicate that this uORF is translated during meiosis [30], but the regulation and roles (if any) of the *SPO24* 5′-extended mRNA, the uORF, and the protein encoded by the dORF (Spo24^d67^) are poorly understood.

Our analyses reveal that in the absence of the dORF, sporulation efficiency decreases significantly, in keeping with the strong induction of this gene during meiosis. Furthermore, the morphology of the sporulated cells is perturbed, with the asci exhibiting an extended shape almost as if the mother cell had begun to enter a “pseudohyphal-like” state during meiosis and sporulation. Intriguingly, the defects are also observed during sporulation of the *spo24*Δ/+ heterozygous strain (though they are less severe than in the homozygous deletion strain), suggesting partial haploinsufficiency. Although the elongated structures are reminiscent of partially pseudohyphal cells, we do not know if this appearance bears any relationship with true pseudohyphal growth. Nonetheless, the relationship between sporulation and pseudohyphal growth is suggestive. Sporulation is induced in yeast through two nutritional conditions: limitation of certain required nutrients, like nitrogen, and the absence of a fermentable carbon source [4], [45]–[48]. When nitrogen is limited in the presence of a fermentable carbon source like glucose, the yeast cells do not sporulate, but rather form pseudohyphae to scavenge further for nutrients [49]. The phenotypes suggest that reduction or loss of Spo24^d67^ expression could affect this balance and perhaps steer the cells away from the meiotic program and toward pseudohyphal development. Extensive additional studies will be required to test this possibility.

The protein encoded by the dORF is expressed throughout meiosis, and appears to exist in both phosphorylated and unphosphorylated states. The balance between these states changes during meiosis, with the highest degree of phosphorylation during mid-meiosis. Among related yeasts, the sequence of Spo24^d67^ is well conserved, especially in the N-terminal half of the protein. The proposed phosphorylation sites are among the conserved residues and match known target consensus sequences for kinases of the GSK3 and PKA families. Ser32 and Ser59 are located within perfect consensus target sequences for PKA enzymes, while serine 63 matches the priming pattern needed for GSK3-mediated phosphorylation. Intriguingly, this family of kinases has previously been implicated with a role in spore formation through the regulation of acetate metabolism [50].

Given the sporulation defect caused by loss of Spo24^d67^ expression, it is striking that Spo24^d67^ localizes to the prospore cortex, as determined by its colocalization with Spo20^51–91^. Spo20 is part of a t-SNARE complex that aids in initial formation of the prospore by driving together the vesicle membranes that form the eventual spore membrane [51]. While the exact role for Spo24 in spore formation remains unknown, its temporal and physical coexpression with Spo20 suggests possible roles in initiating prospore membrane formation.

Intriguingly, the 5′-extended form of the *SPO24* mRNA appears to be important for efficient sporulation, given that specific reduction in 5′-extended mRNA expression from a plasmid (accomplished by MSE deletion) renders that plasmid less effective at rescuing the sporulation defect caused by a *spo24* deletion. These results can be reconciled if the uORF serves a regulatory function, perhaps by modulating Spo24^d67^ translation. Loss of the 5′-extended form via MSE deletion does not reduce the overall accumulation of dORF-containing mRNA, suggesting that the mid-meiotic induction does not serve simply to increase overall *SPO24* mRNA expression levels above a functionally important threshold. Although uORFs are commonly thought to inhibit translation of dORFs by reducing access of scanning ribosomes to the dORF's initiation codon, our results with luciferase reporter constructs, as well as ribosome profiling data [30], support a modestly positive role for the *SPO24* uORF in promoting dORF translation, which could account for the phenotypic effects of MSE deletion. At present we cannot rule out the possibility that the Spo24^u14^ peptide itself exerts a biochemical function, though future studies will be required to address this issue. The ribosome profiling work [30] found many uORFs whose translation is positively correlated with dORF translation, though the mechanisms involved are unknown. Our results support the inclusion of the *SPO24* uORF as a member of this category.

In summary, we have identified *SPO24* (formerly *YPR036W-A*) as a previously unrecognized sporulation gene that encodes a 67-amino-acid phosphoprotein that can localize to the prospore cortex. Loss of Spo24^d67^ function leads to defects in sporulation efficiency as well as ascus morphology. Further delineation of this protein's roles in sporulating cells promises to reveal novel aspects of the meiotic program in yeast.

## Materials and Methods

### RNA Isolation and Real-time Reverse Transcription–Polymerase Chain Reaction (RT-PCR)

mRNA levels were determined using SYBR Green–based quantitative PCR. RNA was extracted using hot phenol chloroform extraction. Briefly, cells were resuspended in AE buffer (50 mM NaOAc, 10 mM EDTA, pH 8.0) with 1.7% SDS. An equal volume of acid phenol:chloroform (5∶1) was added along with zircon/silica beads, vortexed in the cold for 2 minutes, and then alternated between heating at 65°C for 2 minutes and vortexing at 4°C for 2 minutes for ten cycles. The lysate was spun in a microfuge at full speed for 15 minutes and the supernatant was extracted again without heat. A chloroform clean-up was performed, and RNA was then precipitated with isopropyl alcohol and sodium acetate, washed with 70% ethanol, and resuspended in water.

For cDNA synthesis we used Superscript III (Invitrogen) with 1 µg of RNA and gene-specific reverse primers, according to the supplier's protocol. Real-time PCR was performed using gene-specific primers (Table S1) and SYBR Green. Samples were run in triplicate with a genomic DNA control used in the PCR step. For meiotic time course analyses, primers specific for the *PRP8* mRNA were used for normalization.

### Strains, Media and Plasmids

The yeast SK1 strain DKB98 (*MAT*a/α, *ho::LYS2*/”, *lys2*/”, *ura3*/”, *leu2::hisG*/”, *his4X::LEU2/his4B::LEU2*, *arg4*-NspI/*arg4*-BglII) was graciously provided by Doug Bishop (University of Chicago) and used for sporulation and diploid analysis. The haploid sister strain DKB83 (*MAT*a, *lys2*, *ho::LYS2*, *ura3*, *leu2*::*hisG*, *his4X::LEU2*, *arg4*-NspI) was used for haploid analysis. W303-1A (*MAT*a, *leu2-3,112 trp1-1 can1-100 ura3-1 ade2-1 his3-11,15*) was used for luciferase experiments. Yeast strains (Table S2) were constructed by standard genetic crosses or by LiAc transformation [52]. Deletions and chromosomal tags were created using homologous recombination with PCR products created through methods described in Janke *et al.* and Longtine *et al.* [53], [54].

Yeast media including YPD, antibiotic selection plates and synthetic drop-out plates were all made according to standard recipes. Meiosis was induced by shaking cells at 30°C in 50 mL SPM+1/5 COM liquid sporulation medium for 18–48 hours as noted. In the cases where time courses were used, cells were synchronized using pre-sporulation medium containing 10% KOAc following the Bishop lab protocol (http://bishoplab.bsd.uchicago.edu/protocols/SporulationProtocol.pdf) and described previously [29]. Briefly, yeast were grown overnight at 30°C in 5 mL YPD liquid and then diluted into 25 mL of SPS. Cells were shaken for approximately 5 hours, and then diluted for overnight growth to obtain the proper concentration (OD_600_ between 0.5 to 1.4; most cultures were within our preferred range of 0.7–1.0) by the next morning. If cells grew properly they were switched to SPM+1/5 COM medium to begin sporulation. For quantification of sporulation (Figure S2), 5 mL of YPD was inoculated with one colony of each yeast strain to be tested. Three biological replicates of each were cultured. Yeast were shaken at 30°C in YPD overnight then washed with water and then transferred to SPM and shaken again at 30°C for 40 hours. 200 asci from each sample were examined for the number of spores in each, and the average values and standard deviations were calculated. DAPI stains were also performed on cells prepared in the same manner, and the numbers of mononucleate, binucleate, and tetranucleate cells were counted out of 100 unsporulated cells total.

All newly constructed plasmids (Table S3) were confirmed by DNA sequencing. Rescue plasmids were created by first TopoTA-cloning (Invitrogen) a PCR amplicon of *SPO24* from genomic DNA preparations of SK1, including approximately 700 nucleotides (nt) upstream and 400 nt downstream of the annotated ORF. The TA plasmid was then digested with *Eco*RI, and the gel-purified insert was ligated into the *Eco*RI site of the pRS316 vector to yield pSH101. The GFP-tagged derivative, pSH112, was generated by gap repair with a GFP PCR amplicon made with primers that included the appropriate *SPO24* sequences. The PCR product was co-transformed into W303-1A along with *Brs*GI-digested pSH101, and then selected on -Ura plates to yield plasmid pSH112. The plasmid was then extracted and propagated in *E. coli* before transformation into SK1. pSH110 (a pSH101 derivative carrying deletions of the two MSEs) was created by site-directed mutagenesis.

FL' and yCP22FL1 plasmids [38] were kindly provided by John McCarthy (University of Manchester). The luciferase plasmid yCP22FL1 uORF was constructed by cloning in a PCR fragment of *SPO24*'s uORF, along with 512 nt upstream and 7 nt downstream of the uORF, into the *Bam*HI- and *Nde*I-digested yCP22FL1 plasmid. yCP22FL1 uORF^MUT^ was created through site-directed mutagenesis, altering the native AUG start codon of the uORF to a CUG codon.

### Western Blotting and Phosphatase Assay

Protein was extracted from the samples through a TCA protein preparation. Briefly, cell pellets from 5 mL of sporulated culture were resuspended in 300uL of 10% cold TCA, chilled for 10 min on ice, re-centrifuged, and washed with 1 mL of acetone. The pellet was dried in a laminar flow hood for 2 hours and then resuspended in 150 uL protein breaking buffer [50 mM Tris-HCl (pH 7.5), 1 mM EDTA and 2.7 mM DTT]. The sample was vortexed with glass beads in the cold room for 15 minutes with one-minute pauses on ice every 2 minutes. Concentrations were determined using the BioRad Protein Assay. 25 µg of protein was loaded for each sample onto a 15% SDS polyacrylamide gel. A 1∶5000 dilution of anti-myc antibody 9E10 (Santa Cruz Biotechnology) and a 1∶15000 dilution of donkey anti-mouse IR800 secondary antibody (LI-COR Biosciences) were used to visualize myc-tagged Spo24^d67^. Anti-Nap1 loading control antibody (1∶2000) was generously provided by Doug Kellogg (University of California, Santa Cruz). Donkey anti-rabbit IR680 (1∶20,000; (LI-COR Biosciences)) was used as the secondary antibody for Nap1 detection. Blots were imaged on an Odyssey instrument.

Phosphatase treatment consisted of addition of lambda phosphatase and the supplied buffer (New England Biolabs) to 25 µg of protein from the previous extraction. After a one hour incubation at 37°C, samples were loaded onto a 15% SDS polyacrylamide gel and blotted/imaged as above. Mock treatments containing only protein and buffer were done in parallel.

### Luciferase Assays of Translation Efficiency

FL' and yCP22FL1 plasmids [38] [kindly provided by John McCarthy (University of Manchester)] and their derivatives, yCP22FL1 uORF and yCP22FL1 uORF^MUT^, were transformed into W303A and selected on -Trp plates. Positive colonies were grown overnight in 5 mL of -Trp media. The following day 1 mL of overnight culture was diluted into 25 mL -Trp media and were grown exponentially until A_600_ = 0.8 to 1.0. 5 mL of cells were then harvested for preparation of protein extracts for luciferase analysis using the Promega reporter system. Another 10mL was also harvested for total RNA extraction and qRT-PCR as described above, except that *ACT1* mRNA was used for normalization. All analyses were done with three technical replicates. In each case, translational efficiency was calculated as the ratio of translation (represented by luciferase expression) to RNA levels (obtained by qRT-PCR).

### Microscopy and Image Analysis

Fluorescence and differential interference contrast microscopy was performed using a Zeiss Axiovert 200 M microscope equipped with a Cascade II-512B camera (PhotoMetrics, Inc.). Images were taken with a 100×/1.45-numerical aperture oil immersion objective. Images were acquired using the Openlab software (v5.5.0; Improvision).

## Supporting Information

Figure S1 **Confirmation of a 5′-extended** ***YPR036W-A*** **mRNA.**
(TIFF)

Figure S2 **Growth is not inhibited in the** ***ypr036w-aΔ*** **strain.**
(TIFF)

Figure S3 **DAPI staining of** ***ypr036w-aΔ*** **reveals that the majority of unsporulated cells are tetranucleate.**
(TIFF)

Figure S4 **Growth in YPD of WT,** ***spo24Δ/+*** **heterozygous strain and** ***spo24Δ*** **diploid strain shows signs of an elongated, pseudohyphae-like morphology during vegetative growth, consistent with the detectable levels of** ***SPO24*** **expression (** **Figure 1B** **and Figure S1A).**
(TIFF)

Figure S5 **Figure S5: A and B. Deletion of the MSEs decreases expression of the longer (5′-extended) mRNA form.**
(TIFF)

Figure S6 **C-terminal Spo24d67 fusions support wild-type sporulation efficiencies.**
(TIFF)

Figure S7 **Western blot detection of Spo24^d67^-9myc.**
(TIFF)

Figure S8 **Spo24^d67^ conservation in related yeasts.**
(TIFF)

Table S1 **Primers used in this study.**
(TIFF)

Table S2 **Yeast strains used in this study.**
(TIFF)

Table S3 **Plasmids used in this study.**
(TIFF)
