# Supplementary figures and images for: SPO24 Is a Transcriptionally Dynamic, Small ORF-Encoding Locus Required for Efficient Sporulation in Saccharomyces cerevisiae
Source: PLoS One. 2014 Aug 15;9(8):e105058. doi: 10.1371/journal.pone.0105058 (PMC4134269; doi:10.1371/journal.pone.0105058)

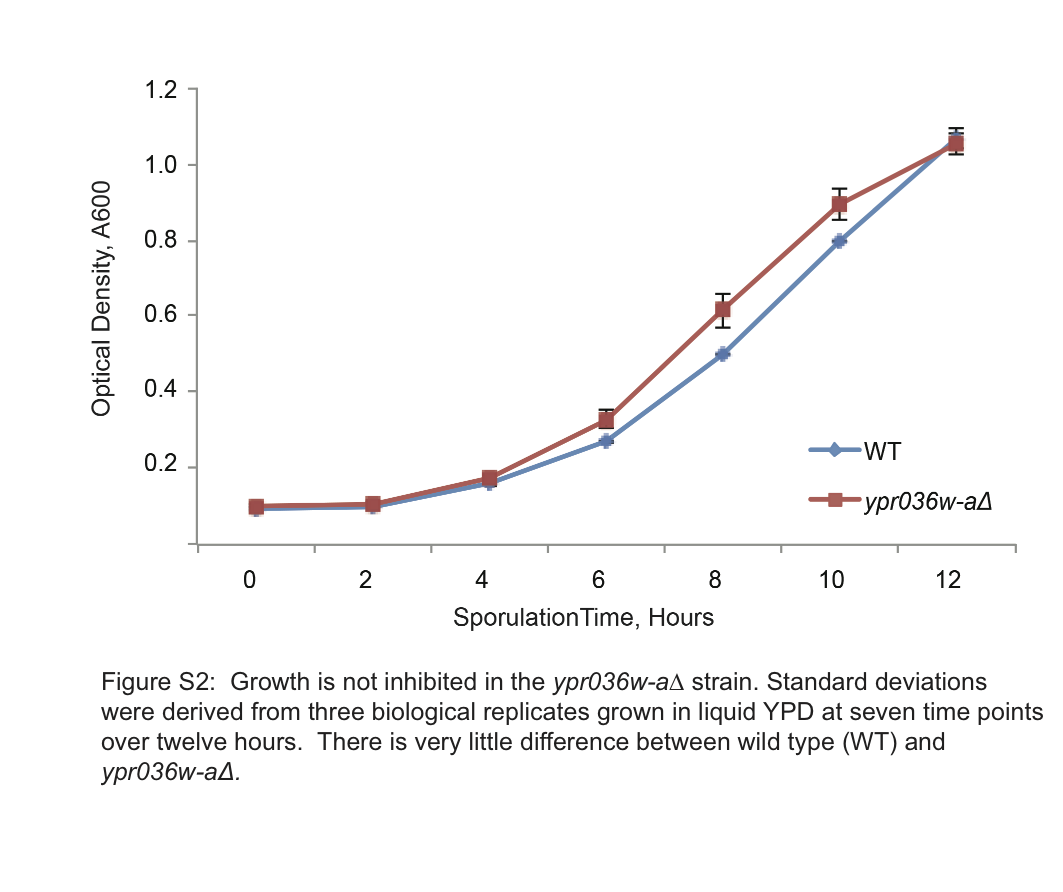

Supplement: Figure S2 — Growth is not inhibited in the ypr036w-aΔ strain. (TIFF) [file pone.0105058.s002.tiff]

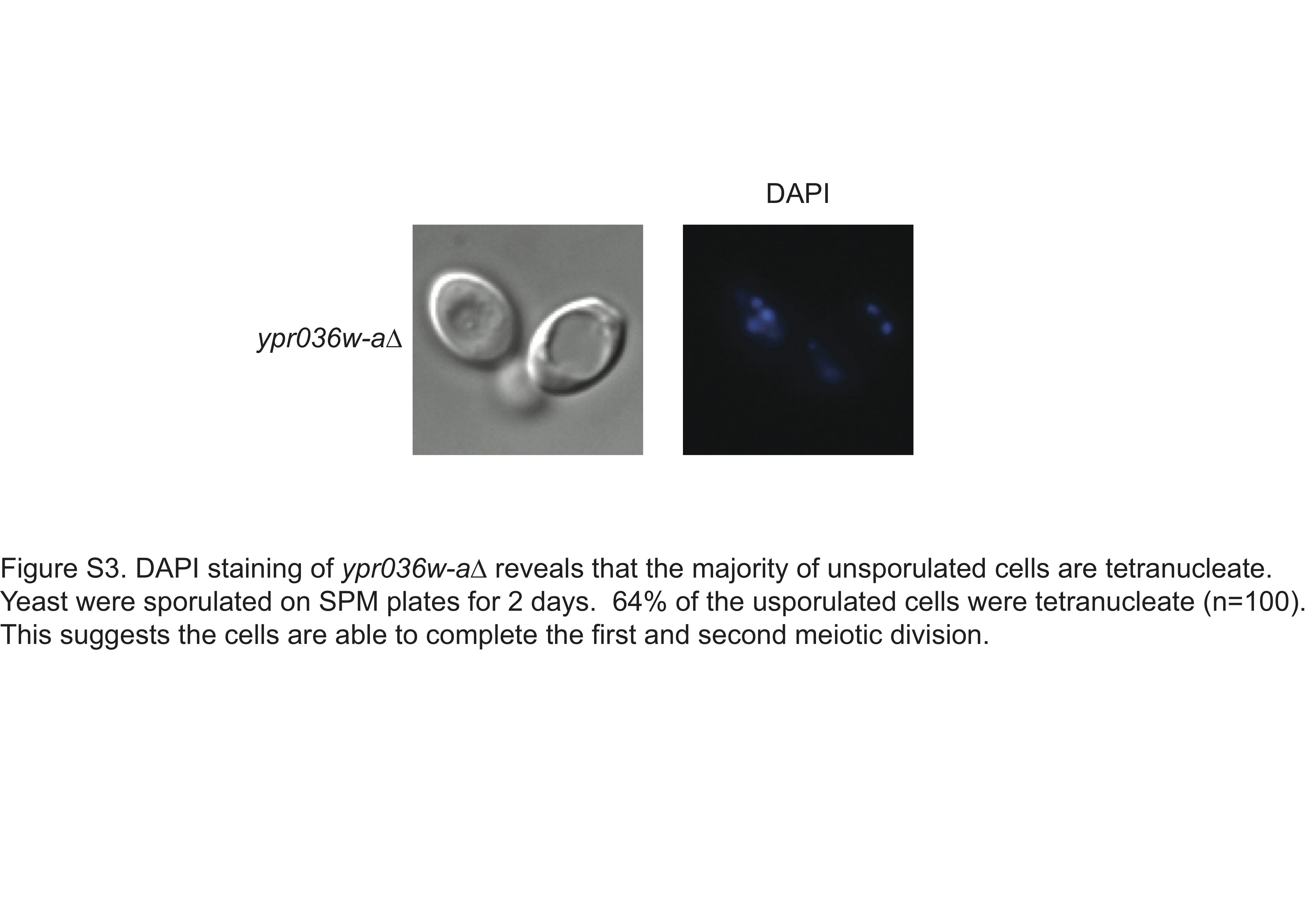

Supplement: Figure S3 — DAPI staining of ypr036w-aΔ reveals that the majority of unsporulated cells are tetranucleate. (TIFF) [file pone.0105058.s003.tiff]

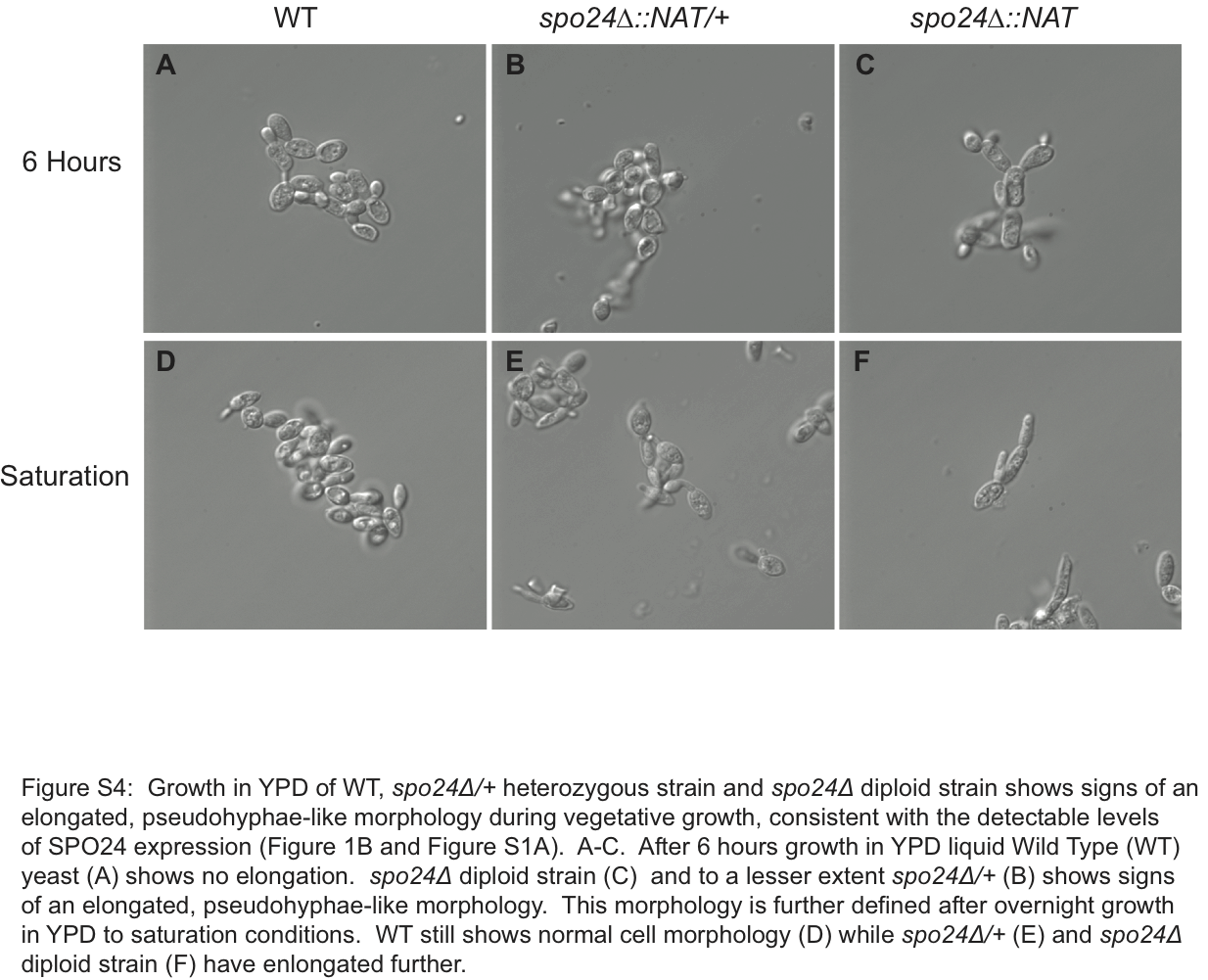

Supplement: Figure S4 — Growth in YPD of WT, spo24Δ/+ heterozygous strain and spo24Δ diploid strain shows signs of an elongated, pseudohyphae-like morphology during vegetative growth, consistent with the detectable levels of SPO24 expression ( Figure 1B and Figure S1A). (TIFF) [file pone.0105058.s004.tiff]

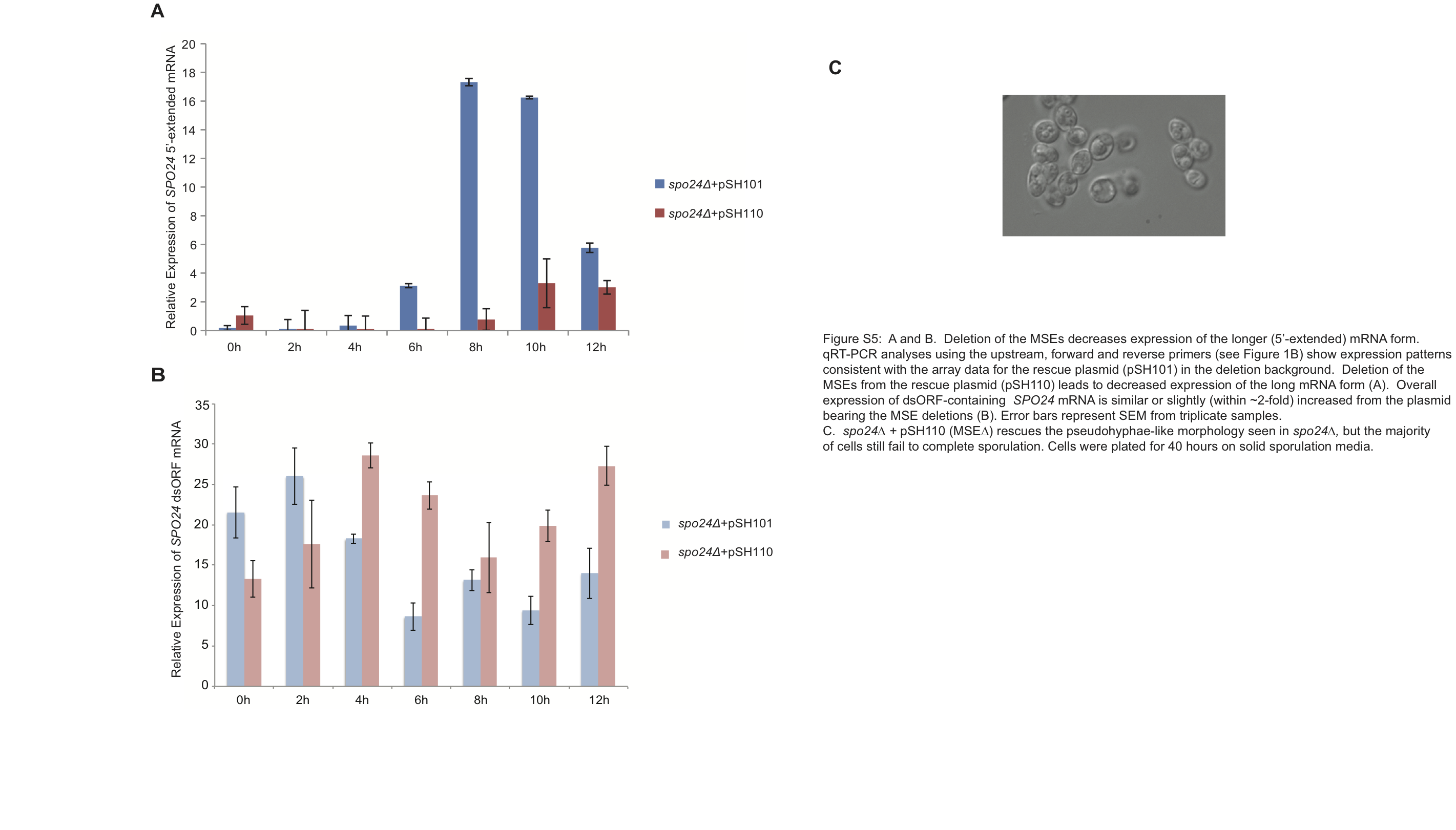

Supplement: Figure S5 — A and B. Deletion of the MSEs decreases expression of the longer (5′-extended) mRNA form. (TIFF) [file pone.0105058.s005.tiff]

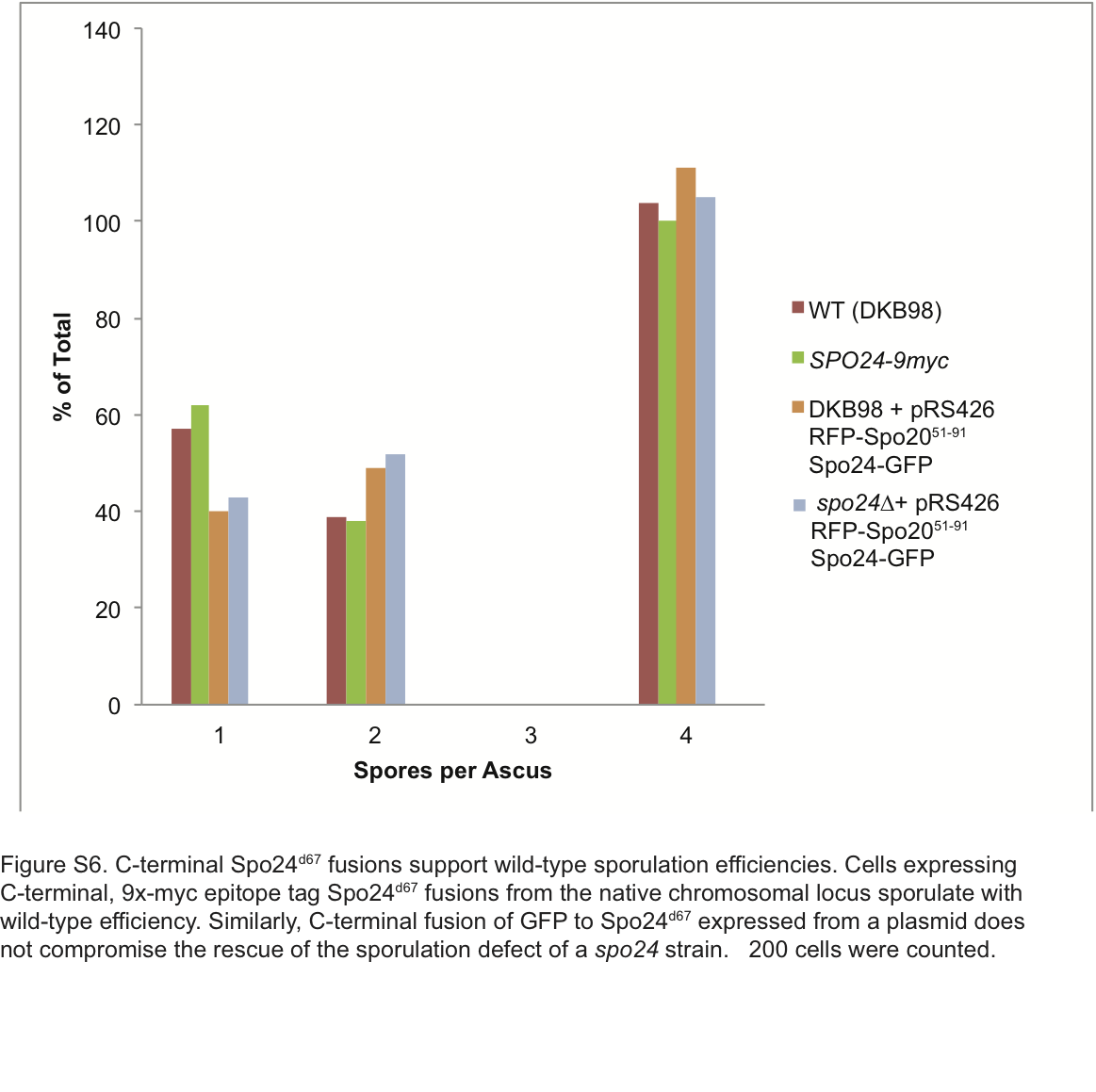

Supplement: Figure S6 — C-terminal Spo24d67 fusions support wild-type sporulation efficiencies. (TIFF) [file pone.0105058.s006.tiff]

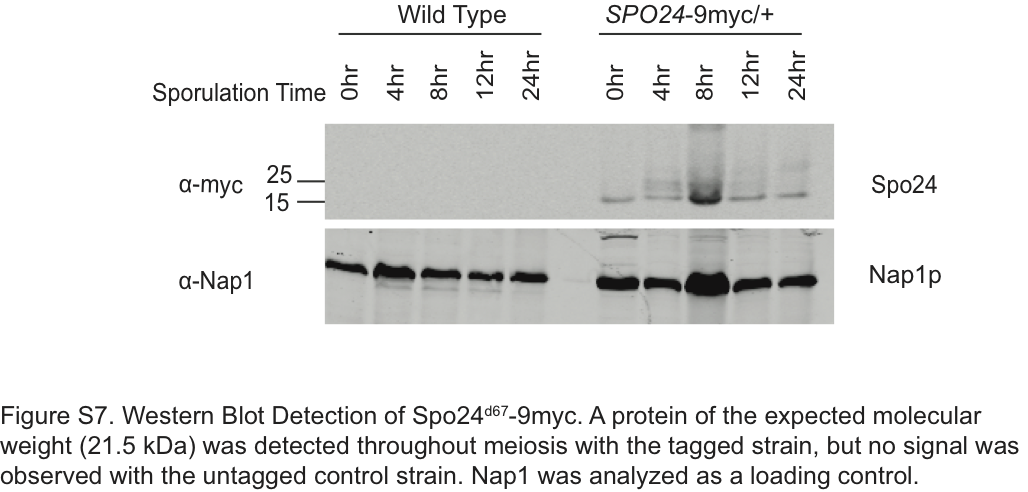

Supplement: Figure S7 — Western blot detection of Spo24d67-9myc. (TIFF) [file pone.0105058.s007.tiff]

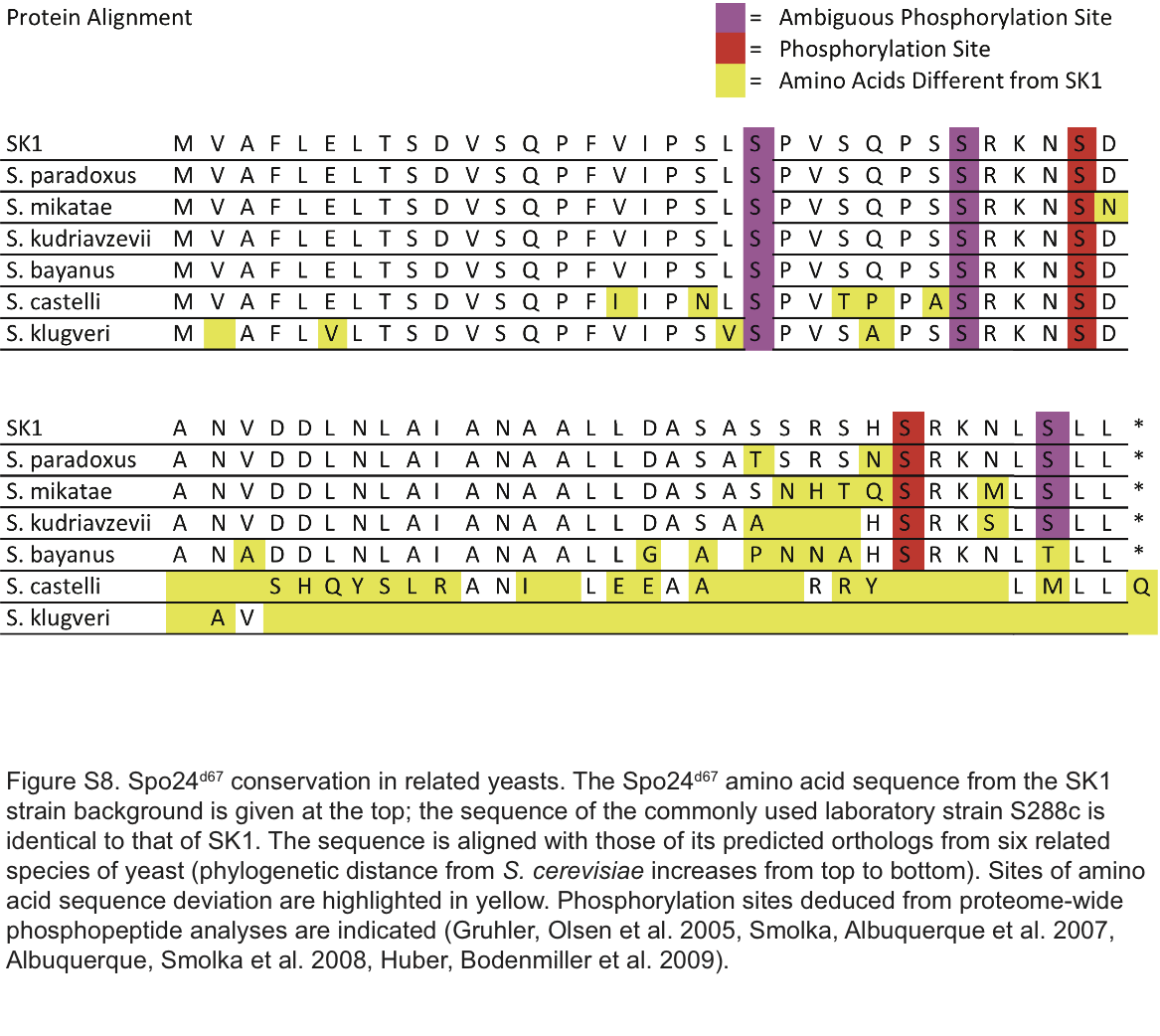

Supplement: Figure S8 — Spo24d67 conservation in related yeasts. (TIFF) [file pone.0105058.s008.tiff]

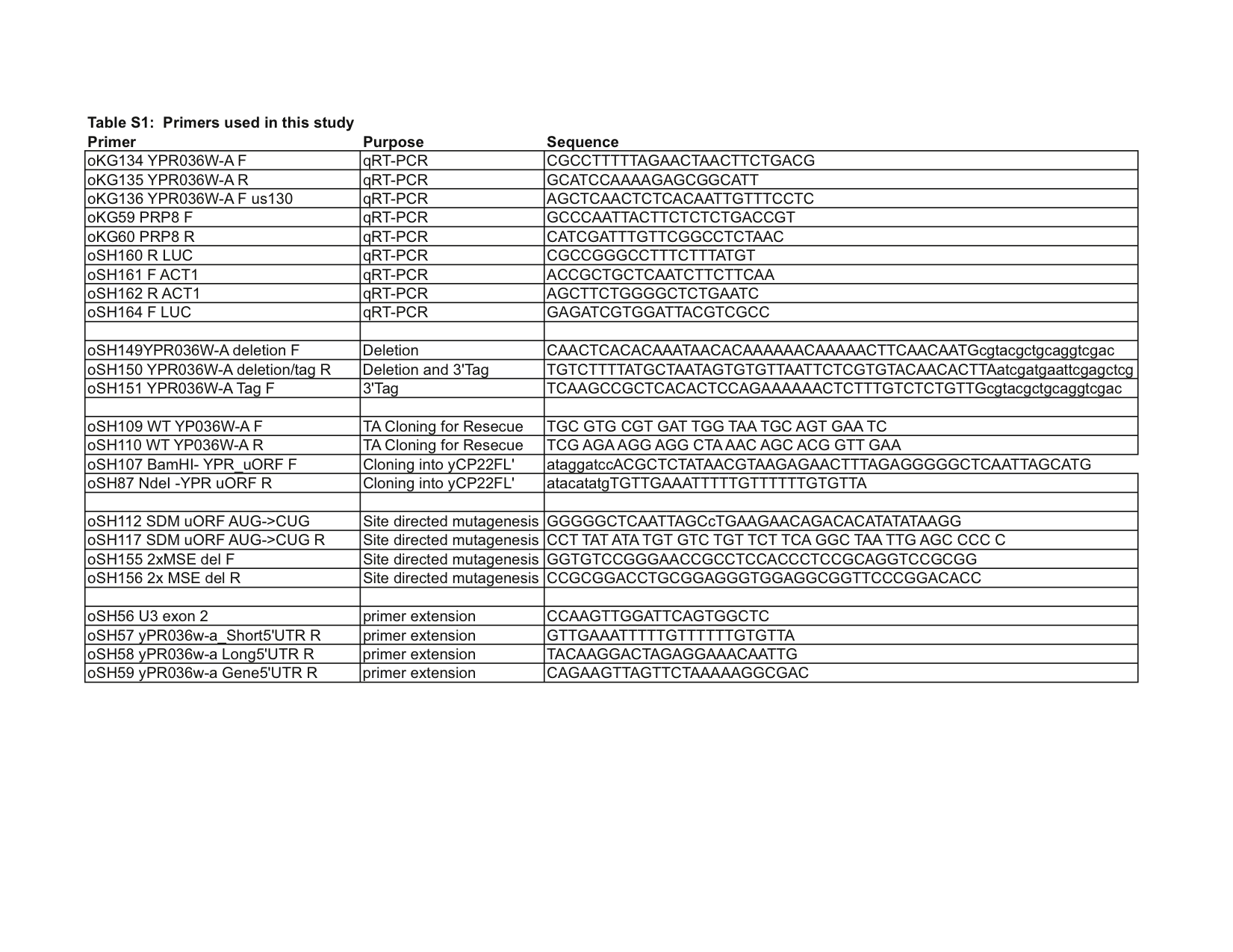

Supplement: Table S1 — Primers used in this study. (TIFF) [file pone.0105058.s009.tiff]

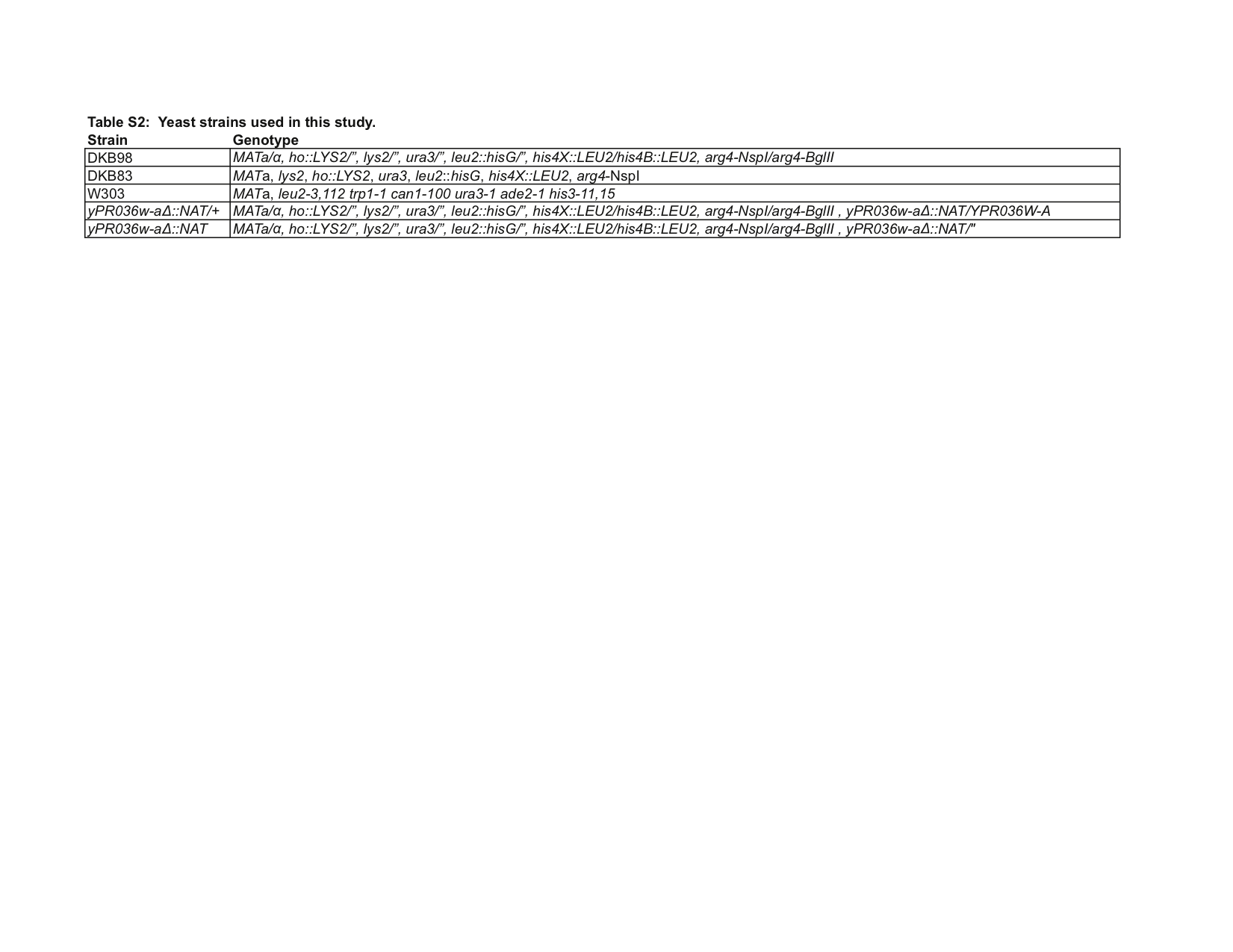

Supplement: Table S2 — Yeast strains used in this study. (TIFF) [file pone.0105058.s010.tiff]

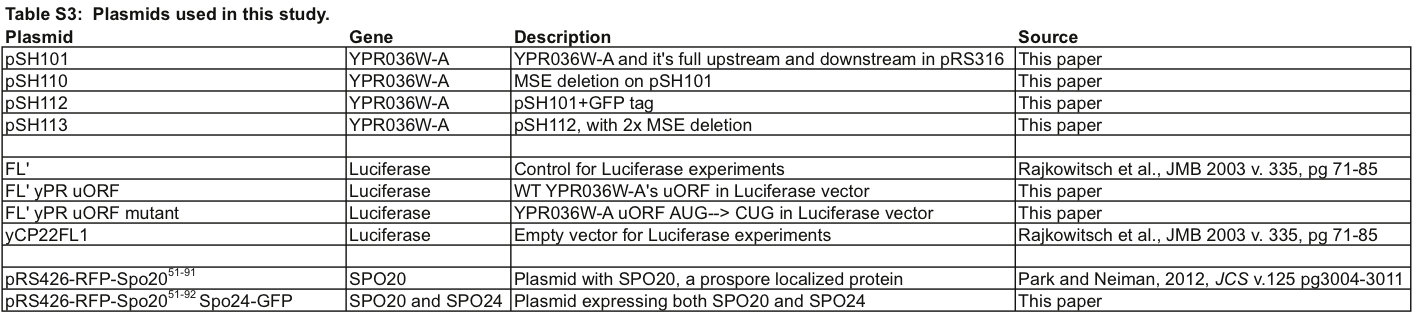

Supplement: Table S3 — Plasmids used in this study. (TIFF) [file pone.0105058.s011.tiff]
